# A continuous fall of PSA use for prostate cancer screening among Brazilian doctors since 2001. Good or bad notice?

**DOI:** 10.1590/S1677-5538.IBJU.2018.0179

**Published:** 2019-07-27

**Authors:** Fernando Antônio Glasner da Rocha Araújo, Nairo Massakazu Sumita, Ubirajara de Oliveira Barroso

**Affiliations:** 1 Departamento de Medicina, Faculdade de Medicina da Universidade Federal da Bahia, Salvador, BA, Brasil;; 2 Divisão de Química Clínica, Fleury Medicina e Saúde, São Paulo, SP, Brasil;; 3 Departamento de Cirurgia Especial, Faculdade de Medicina da Universidade Federal da Bahia, Salvador, BA, Brasil;; 4 Escola Bahiana de Medicina e Saúde Pública, Salvador, BA, Brasil

**Keywords:** Prostatic Neoplasms, Prostate-Specific Antigen, Mass Screening

## Abstract

**Purpose:**

To evaluate the trend of use of Prostate Specific Antigen (PSA) for screening of prostate cancer (PC) among Brazilian doctors, from the beginning of its regular availability in clinical laboratories.

**Material and Methods:**

A serial cross-sectional study was performed using data obtained from a large database between 1997 and 2016. The general PSA screening trend during this period, adjusted for the total number of exams performed in men, was analyzed. Time-series analysis was performed through observation of the general regression curve using the generalized least squares method, and the impact of the recommendations was assessed with autoregressive integrated moving average (ARIMA) models.

**Results:**

During the period studied 2,521,383 PSA determinations were done. The age of the participants ranged from 21 to 111 years, with an average of 56.7 ± 22.7 years. The relative number of PSA tests/100.000 exams in males showed a constant reduction since 2001, and this trend was more evident in the group aged 55-69 years. Although statistically significant, the impact of reduced PSA screening after the 2012 USPSTF publication was clinically irrelevant.

**Conclusions:**

Our results indicated a continuous reduction in the use of PSA screening over time, regardless of the publication of recommendations or clinical guidelines. The fact that this trend was more pronounced among those with a greater benefit potential (55-69 years), relative to groups with a greater damage potential due to overdiagnosis and overtreatment (aged >74 years and <40 years), is a matter of concern. Follow-up studies of these trends are advisable.

## INTRODUCTION

In Brazil, as in other countries, prostate cancer (PC) is the second most common type of cancer in men, with an estimated risk of 70.42 new cases per 100,000 men (1). For many years, screening for early detection of prostate cancer relied solely on digital rectal examination. During this period, most cancers were diagnosed in advanced stages, with no impact on mortality reduction. The introduction of the prostate-specific antigen (PSA) screening test resulted in a dramatic increase in early-stage PC diagnoses, followed by a reduction in mortality (2). These trends prompted several scientific societies to recommend PSA screening for early diagnosis of PC.

Following the publication of large, randomized trials (3, 4) and subsequent recommendations after 2009, several studies have examined the impact of these recommendations on the frequency of PSA screening among individuals in the United States (5–12), France (13), and Sweden (14), albeit with conflicting results. In particular, the recommendations against prostate cancer screening (PCS) of the United States Preventive Services Task Force (USPSTF) have been assessed among the elderly in 2008 (15), and men in general in 2012 (16).

In Brazil, the National Cancer Institute (INCA) published a technical note, in November 2013, that recommended against offer of screening by the doctor (17). The Brazilian Society of Urology recommends that during routine consultations doctors should discuss the possibility of screening with their patients (opportunistic screening) informing them of the controversies, possible risks and benefits, in order to make a shared decision (18).

Recently, the USPSTF drafted a recommendation changing its previous grade D rating to a C rating for men between 55 and 69 years. This decision process collectively considered the benefits and risks involved (19). The impact of this publication has not yet been evaluated.

Currently, there are no time-series studies related to the use of PSA screening in Latin America. Thus, this study sought to identify PSA screening trends using historical series data from a large national laboratory, and evaluate the possible impact of the USPSTF (16) and INCA (17) recommendations on PSA screening in Brazil.

## MATERIALS AND METHODS

This study was approved by the Research Ethics Committee of the authors’ institution (CAAE 55705116.6.0000.5544). A serial cross-sectional study was performed using a private laboratory database of national scope in Brazil. The following data were collected from October 1997 (marking the start of PSA screening in this laboratory) until the end of December 2016: exam date, city where the data was obtained from, patient age, physician specialty, and PSA screening result. In addition, the total number of exams, the total number of cholesterol in men and women, and the total number of PSA exams were collected monthly and for each age group.

Since it was impossible to determine the reason behind the exams requests, namely, whether exams were for screening or diagnostic purposes, certain measures were adopted to reduce the possibility of confounding results. To minimize the possibility of including diagnostic requests, and given that most patients who receive follow-up exams undergo more than one exam request per year (20), patients with more than one exam request per year were excluded. This measure was similar to, albeit more restrictive than that adopted by other studies (21).

To account for changes associated with population growth, the expansion of the sample collection points and the possible seasonality of the tests, we established a rate “number of PSA / 100,000 tests performed in men” per month. We tried to directly extract the total number of tests performed in men for each month, however, because they were very robust numbers, it was not possible, operationally, to obtain it directly. Thus, using the hypothesis that the number of total cholesterol tests is proportional to the total number of tests performed each month, we multiplied the total number of tests performed each month by the percentage of cholesterol tests performed by men, obtaining an estimate of the total number of exams performed by men each month.

The strategy of using the number of cholesterol tests performed per period to minimize seasonality was previously used by Aslani et al. (8). These authors observed the PSA utilization curve, from a ratio “N exams of PSA/N exams of cholesterol”. However, because the number of cholesterol requests could be influenced, for example, by heart disease prevention campaigns over the years, this could distort the PSA utilization curve. Thus, we chose to use the number of cholesterol performed by men only as a factor of monthly distribution of the total number of tests performed by men per month.

In addition to the global population, the utilization of PSA screening tests in specific age groups was considered:

1. Group I comprised individuals aged between 55 and 69 years; the only group for which there is an universal acceptance of a screening benefit (22).
2. Group II comprised individuals aged >74 years; whether or not screening should be performed for this age group remains controversial (23).
3. Group III comprised individuals aged <40 years; universally accepted as having a very low probability of benefiting and a high probability of damage resulting from screening (23).

The possible impact of two publications, namely, the 2012 USPSTF recommendation positing against screening at any age (16), and the technical note issued by INCA in 2013 advising against regular screening (17), was analyzed for all groups.

For the analysis of the time series, two approaches were adopted: in the first, a general regression line was estimated through linear model using generalized least squares to get an idea of the trend of the series in time. In the second approach, to measure the effect of intervention, integrated dynamic regression models (ARIMA) with assumed two permanent change interventions (occurred in 2012 and 2013), following the Box-Jenkins methodology were used, as they allow for the incorporation and adjustment of the effect of a historical series autocorrelation, reducing such bias when estimating trends. For all series studied, those that were non-stationary were differentiated. Then, structural and seasonal parameters of auto-regression and moving averages were estimated (autocorrelation (AR), differentiation (d), moving average (MA)), with an ARIMA notation (AR, d, MA) (AR,d,MA)S, as well as the slopes of the regressions (β) representing the changes in average trends of the series, per year. To diagnose the best model, the Akaike's information criterion that provided the least value was obtained for each series, together with the residual analysis, observation of autocorrelation and partial autocorrelation graphs (descriptively through the Ljung-Box test), evaluation of parameter overestimation, and comparison of the original data with those predicted by the models. Since the entire target population was studied, inferential statistics were not calculated, but p values were used descriptively. The statistical package R3, release 3.3, was used for the analysis of data in this temporal series.

## RESULTS

From October 1997 to December 2016, 2,521,283 PSA exams and 172,474,779 total exams were performed in men. Table-1 shows the annual number of PSA and total exams in males (TM), as well as the frequency-adjusted PSA exam rates.

**Table 1:** Number of total and relative PSA tests per year.

Year	PSA tests	TM	PSA/100,000 TM
1997	7.008	380.245	1.843
1998	36.246	1,723.981	2.102
1999	39.267	1,830.866	2.145
2000	44.231	1,910.617	2.315
2001	47.371	2,087.817	2.269
2002	48.793	2,338.036	2.087
2003	51.780	2,541.588	2.037
2004	52.578	2,591.503	2.029
2005	54.006	2,741.804	1.970
2006	56.350	3,043.925	1.851
2007	60.355	3,407.954	1.771
2008	71.583	4,256.155	1.682
2009	123.918	7,423.250	1.669
2010	182.329	10,481.023	1.740
2011	270.628	14,647.501	1.848
2012	260.397	16,736.905	1.556
2013	285.857	20,877.515	1.369
2014	261.833	22,309.558	1.174
2015	262.913	24,963.972	1.053
2016	303.940	26,180.563	1.161

**TM** = Total exams in males

The age of the participants ranged from 21 to 111 years, with an average of 56.7±22.7 years (Table-2).

**Table 2:** Age distribution of men undergoing PSA tests.

Age (years)	PSA	
	N	%
<40	158.712	6.29%
40-44	229.870	9.12%
45-49	315.988	12.53%
50-54	366.274	14.53%
55-69	947.161	37.57%
70-74	208.208	8.26%
>74	288.889	11.46%
ND	6.281	0.25%
**Total**	**2,521.383**	**100.00%**

**Figure 1:**
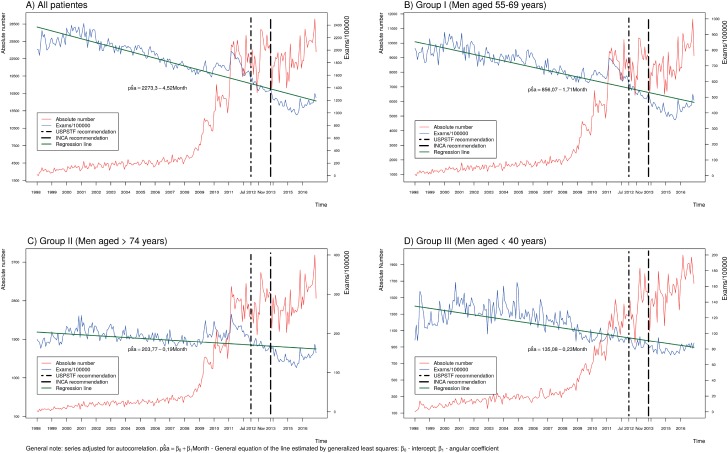
Use of PSA for Prostate Cancer Screening, Brazil, 1997-2016.

The PSA utilization curve increased after 2009, consistent with the expansion of the number of units in the laboratory (line red in Figure-1A). However, the relative number of PSA tests/100.000TM (line blue in Figure-1A) showed a constant reduction, with two periods of short rising (1997-2001 and 2010-2011). A similar pattern was observed for all age groups studied, but was more pronounced in group I (Figure-1B) than in groups II and III (respective regression coefficients of −1,71; −0,9; −0,23).

The analysis of the impact of the interventions (USPSTF and INCA publications) showed a reduction in the overall use of PSA screening (Table-3). The reduced PSA screening trends among total individuals and in Group I (55-69 years), as well as increased screening trends in the “elder” (Group II, >74 years) and “young” (Group III <40 years) groups, remained consistent after the publication of the INCA technical note (November 2013). Although statistically significant, the effects of these publications were limited in absolute terms.

**Table 3:** Analysis of the impact of the USPSTF (2012) and INCA (2013) recommendations.

Series	Estimated Model	AIC	From July 2012		From November 2013	
			ω* (p value)	δ** (p value)	ω* (p value)	δ** (p value)
Total	ARIMA (6,1,0)(8,1,0)_12_	2,585.9	113.67 (0.0326)	-0.62 (<0.0001)	-9.11 (0.9271)	-0.32 (0.9871)
<40 years	ARIMA (4,1,0)(11,0,0)_12_	1,713.9	4.98 (0.1464)	0.65 (<0.0001)	4.26 (0.1879)	0.68 (<0.0001)
55 – 69 years	ARIMA (5,1,0)(8,1,0)_12_	2,246.9	30.32 (0.2703)	0.12 (0.8611)	-23.21 (0.2721)	-0.69 (0.0052)
>74 years	ARIMA (6,1,0)(9,0,0)_12_	1,801.1	2.65 (0.0070)	0.94 (<0.0001)	3.30 (0.1400)	0.90 (<0.0001)

ω * Estimated parameter /100,000 tests according to the model: magnitude of the effect after the intervention

δ ** Rate of monthly change after the intervention (progression)

**AIC** = Akaike Information Criterion - adjustment of the model without transfer function

## DISCUSSION

Our results indicate a reduction in the utilization of PSA tests for prostate cancer screening during the observation period despite publication of the two recommendations analyzed.

Several studies have addressed the impact of recommendations on the frequency of PSA screening. However, none of these studies covered a period as long as that assessed here. Indeed, most of these studies covered periods close to the events of interest.

Several authors found a reduction in PSA screening, similar to our study. For example, Aslani et al. (8) found a significant reduction in PSA screening after 2009, which intensified, albeit not significantly, after the 2012 USPSTF recommendation.

On the other hand, Drazer et al. (9) observed that the percentage of screened men in any age group, including those over 75 years of age, did not change between 2005 and 2010. However, a significant reduction in the number of individuals belonging to the age group most likely to benefit from screening (>50 years) was observed between 2010 and 2013, consistent with the trends reported herein.

In France, Eisinger et al. (13) observed an increase in screening between 2005 and 2008, followed by a plateau between 2008 and 2011, with a significant reduction only in individuals with higher income (13). Although we have not evaluated the economic level of our cases, our sample is composed mainly of patients who have health insurance, which in Brazil are usually from higher economic extracts.

The only age group that did not show a clear reduction in screening rates in our study was the elder group (>74 years), which exhibited relative stability. Many previous studies demonstrated a reduction in PSA screening after this age (5, 6, 9, 10), although some reported no differences (11, 24) and others observed an increase in the number of PSA requests among these patients (14).

The discrepancies among these studies can be partially explained by differences in sampling. Institutions, which are capable of influence medical activity, are more likely to follow protocols, such as Medicare (6, 7), Veterans Health Administration (5), and patients in managed care (10) or health insurers (11). On the other hand, studies based on interviews that rely on patients’ memories may have lower accuracy (25) and be less likely to exhibit differences (24). Another factor that may play a role, at least in the population above 75 years of age, is the country of origin. A greater adherence to recommendations has been observed in North America (5–7, 9, 10) compared to other countries (13, 14, 20), including the present study in Brazil.

One limitation common to all of the cited studies is the question of whether any trends observed shortly after events are truly associated with these events. There is no simple way to predict the time window between the publication of a recommendation and a possible change in a physician's attitude. The clinical implementation of research is certainly slow (26), and any utilization trends cannot be immediately attributed to publications. Neither this, nor any study cited herein, directly questioned the physicians or patients on whether their decision to request or perform an PSA request was influenced by the recommendations of governmental or scientific institutions.

The strengths of this study include the sample size; representation of the entire period of PSA testing, beginning from the earliest days of the test's commercial use in Brazil; and the broad coverage area, ranging from the poorest (northeast) to the richest regions (south and southeast) of the country.

The large increase in the number of laboratory units that occurred after 2009 revealed a large increase in the absolute number of PSA tests, however, as we observed the trend curve relativized by the number of cholesterol tests, any distortion caused by the rapid growth of the laboratory should have been offset by similar impact on the number of cholesterol tests.

An important limitation of this and other (10, 21, 24) studies was that, despite adopted measures, it was impossible to determine with certainty the reason behind exam requests, i.e., screening or diagnosis. Nonetheless, although this uncertainty may result in an increase in the number of requests, this increase was likely constant over the different periods and therefore unlikely to influence the trend of the exam utilization curve. For instance, if the number of screening tests was overestimated in the period from 1997-2001, it is likely that this number was similarly overestimated in other periods. Therefore, since we assessed utilization curve trends, any biases that may have occurred were presumably small (27).

Another limitation concerns the fact that most patients screened for PSA have health insurance. As a result, it is difficult to generalize our results to populations without health insurance. However, in Brazil, the Unified Health System (SUS) provides assistance to all individuals, albeit with some limitations. In fact, the health care units of the SUS are required by law to perform tests for the early detection of prostate cancer upon physician request (28).

## CONCLUSIONS

Our results indicate a continuous reduction in PSA screening over time, regardless of the publication of recommendations or clinical guidelines. This finding may indicate a learning curve, with desirable results, such as reduced probabilities of overdiagnosis and overtreatment. However, it is important to perform follow-up studies, especially among those with the greatest probability of benefit (aged 55-69 years), since screening reductions may not be as desirable in this age group. On the other hand, the use of PSA screening for PC in young individuals (below 40 years of age) is of concern, and warrants further studies to understand the origin of the problem and prevent harm associated with undue screening.

## References

[1] INCA (Instituto Nacional do Câncer) Estimativa da Incidência de Câncer no Brasil 2014.

[2] Horner MJ, Ries LAG, Krapcho M, Neyman N, Aminou R, Houlander N SEER Cancer Statistics Review, 1975-2006.

[3] Andriole GL, Crawford ED, Grubb RL, Buys SS, Chia D, Church TR (2009). Mortality results from a randomized prostate-cancer screening trial. N Engl J Med..

[4] Schröder FH, Hugosson J, Roobol MJ, Tammela TL, Ciatto S, Nelen V (2009). Screening and prostate-cancer mortality in a randomized European study. N Engl J Med..

[5] Zeliadt SB, Hoffman RM, Etzioni R, Gore JL, Kessler LG, Lin DW (2011). Influence of publication of US and European prostate cancer screening trials on PSA testing practices. J Natl Cancer Inst..

[6] Ross JS, Wang R, Long JB, Gross CP, Ma X (2012). Impact of the 2008 US Preventive Services Task Force recommendation to discontinue prostate cancer screening among male Medicare beneficiaries. Arch Intern Med..

[7] Howard DH, Tangka FK, Guy GP, Ekwueme DU, Lipscomb J (2013). Prostate cancer screening in men ages 75 and older fell by 8 percentage points after Task Force recommendation. Health Aff (Millwood)..

[8] Aslani A, Minnillo BJ, Johnson B, Cherullo EE, Ponsky LE, Abouassaly R (2014). The impact of recent screening recommendations on prostate cancer screening in a large health care system. J Urol..

[9] Drazer MW, Huo D, Eggener SE (2015). National Prostate Cancer Screening Rates After the 2012 US Preventive Services Task Force Recommendation Discouraging Prostate-Specific Antigen-Based Screening. J Clin Oncol..

[10] Wallner LP, Hsu JW, Loo RK, Palmer-Toy DE, Schottinger JE, Jacobsen SJ (2015). Trends in Prostate-specific Antigen Screening, Prostate Biopsies, Urology Visits, and Prostate Cancer Treatments From 2000 to 2012. Urology..

[11] Rezaee ME, Ward CE, Odom BD, Pollock M (2016). Prostate cancer screening practices and diagnoses in patients age 50 and older, Southeastern Michigan, pre/post 2012. Prev Med..

[12] Sammon JD, Abdollah F, Choueiri TK, Kantoff PW, Nguyen PL, Menon M (2015). Prostate-Specific Antigen Screening After 2012 US Preventive Services Task Force Recommendations. JAMA..

[13] Eisinger F, Morere JF, Touboul C, Pivot X, Coscas Y, Blay JY (2015). Prostate cancer screening: contrasting trends. Cancer Causes Control..

[14] Nordström T, Aly M, Clements MS, Weibull CE, Adolfsson J, Grönberg H (2013). Prostate-specific antigen (PSA) testing is prevalent and increasing in Stockholm County, Sweden, Despite no recommendations for PSA screening: results from a population-based study, 2003-2011. Eur Urol..

[15] U.S. Preventive Services Task Force (2008). Screening for prostate cancer: U.S. Preventive Services Task Force recommendation statement. Ann Intern Med..

[16] Moyer VA (2012). U.S. Preventive Services Task Force. Screening for prostate cancer: U.S. Preventive Services Task Force recommendation statement. Ann Intern Med..

[17] INCA (Instituto Nacional do Câncer) (2013). Rastreamento do Câncer de Próstata 2013.

[18] Dall’Oglio MF, Crippa A, Faria EF, Cavalhal GF, Milfont JC, Jr JP (2011). Rastreamento do câncer de próstata. Diretrizes de Câncer de Próstata.

[19] Bibbins-Domingo K, Grossman DC, Curry SJ (2017). The US Preventive Services Task Force 2017 Draft Recommendation Statement on Screening for Prostate Cancer: An Invitation to Review and Comment. JAMA..

[20] Carmichael LK, Goldsbury DE, O’Connell DL (2013). Prostate cancer screening for men aged 75 to 84 years in New South Wales. Aust N Z J Public Health..

[21] Lee SY, Friderici J, Stefan MS, Rothberg MB (2014). Impact of the 2008 U.S. Preventative Services Task Force recommendation on frequency of prostate-specific antigen screening in older men. J Am Geriatr Soc..

[22] Ilic D, Neuberger MM, Djulbegovic M, Dahm P (2013). Screening for prostate cancer. Cochrane Database Syst Rev..

[23] Kerfoot BP, Holmberg EF, Lawler EV, Krupat E, Conlin PR (2007). Practitioner-level determinants of inappropriate prostate-specific antigen screening. Arch Intern Med..

[24] Scosyrev E, Wu G, Golijanin D, Messing E (2012). Prostate- specific antigen testing in older men in the USA: data from the behavioral risk factor surveillance system. BJU Int..

[25] Hall HI, Van Den Eeden SK, Tolsma DD, Rardin K, Thompson T, Hughes Sinclair A (2004). Testing for prostate and colorectal cancer: comparison of self-report and medical record audit. Prev Med..

[26] Ilic D, Murphy K, Green S (2013). What do general practitioners think and do about prostate cancer screening in Australia?. Aust Fam Physician..

[27] Schröder FH (2013). General practitioner (GP)'s view on screening for prostate cancer in the Netherlands: the impact of a randomized trial. BJU Int..

[28] Brasil (2014). Lei 13.045.

